# Both TLR2 and TRIF Contribute to Interferon-β Production during *Listeria* Infection

**DOI:** 10.1371/journal.pone.0033299

**Published:** 2012-03-14

**Authors:** Camille Aubry, Sinéad C. Corr, Sebastian Wienerroither, Céline Goulard, Ruth Jones, Amanda M. Jamieson, Thomas Decker, Luke A. J. O'Neill, Olivier Dussurget, Pascale Cossart

**Affiliations:** 1 Institut Pasteur, Unité des Interactions Bactéries-Cellules, Paris, France; 2 Inserm, U604, Paris, France; 3 INRA, USC2020, Paris, France; 4 Université Paris Diderot, Sorbonne Paris Cité, Cellule Pasteur, Paris, France; 5 School of Biochemistry and Immunology, Trinity Biomedical Sciences Institute, Trinity College Dublin, Ireland; 6 Max F. Perutz Laboratories, Department of Genetics, Microbiology and Immunobiology, University of Vienna, Vienna, Austria; University of São Paulo, Brazil

## Abstract

Synthesis of interferon-β (IFN-β) is an innate response to cytoplasmic infection with bacterial pathogens. Our recent studies showed that *Listeria monocytogenes* limits immune detection and IFN-β synthesis via deacetylation of its peptidoglycan, which renders the bacterium resistant to lysozyme degradation. Here, we examined signaling requirements for the massive IFN-β production resulting from the infection of murine macrophages with a mutant strain of *L. monocytogenes*, Δ*pgdA*, which is unable to modify its peptidoglycan. We report the identification of unconventional signaling pathways to the IFN-β gene, requiring TLR2 and bacterial internalization. Induction of IFN-β was independent of the Mal/TIRAP adaptor protein but required TRIF and the transcription factors IRF3 and IRF7. These pathways were stimulated to a lesser degree by wild-type *L. monocytogenes*. They operated in both resident and inflammatory macrophages derived from the peritoneal cavity, but not in bone marrow-derived macrophages. The novelty of our findings thus lies in the first description of TLR2 and TRIF as two critical components leading to the induction of the IFN-β gene and in uncovering that individual macrophage populations adopt different strategies to link pathogen recognition signals to IFN-β gene expression.

## Introduction

Detection of microbial pathogens by pattern recognition receptors, such as Toll-like receptors (TLRs) triggers innate immune responses as a first line of defense against infections [1]–[3]. Pathogen-associated molecular patterns (PAMPs) such as bacterial cell walls and their structural components induce a vast variety of biological effects in host organisms. The innate response against infection with intracellular pathogens includes the synthesis of type I IFNs (IFN-I). Whereas this cytokine family generally protects against viruses, its impact on bacterial infections can be either detrimental or advantageous for the host organism [4].

*Listeria monocytogenes* is a bacterial pathogen which replicates in the cytoplasm of infected cells. Cytosolic pattern recognition receptors (PRRs) respond to cytosolic bacterial products and contribute to the induction of the innate immune response [5], [6]. Previous studies in bone marrow-derived macrophages (BMM) and epithelial cells show that in these cell types the synthesis of IFN-I in response to infection with *L. monocytogenes* is independent of TLRs and their adapters, relying exclusively on signals originating from cytosolic sensors [5]–[8]. DNA as well as cyclic dinucleotides released from lysed bacteria were suggested to function as the relevant *L. monocytogenes* PAMPs [9]–[11]. Several cytosolic proteins with the ability to sense pathogen-derived nucleic acids have recently been described [11]–[19]. Cytosolic recognition of *L. monocytogenes* causes the activation of the serine/threonine kinase TBK1 and the phosphorylation of its substrate transcription factors IRF3 and IRF7 [7], [8]. Both IRF3 and IRF7 participate in the formation of an enhanceosome at the IFN-β promoter [20].

During uptake by host cells *L. monocytogenes* is exposed to plasma membrane and endosomal TLRs. Among these, TLR2 which recognizes lipotechoic acids and lipopeptides, contributes to the innate response against infection [21]–[23]. Reportedly, TLR2 signals through the interacting adapter proteins Mal/TIRAP and MyD88 and does not contribute to the synthesis of type I IFN in *Listeria*-infected BMM [7]–[9]. Signaling through TRIF, an adapter protein known to connect TLRs 3 and 4 with the IFN-I genes was similarly ruled out for *Listeria*-infected BMM [7].

In order to establish a successful infection, pathogens must survive host defense systems or else mitigate the activities of PRRs. Consequently, they have evolved to modify the structural components which normally trigger PRR responses. Bacterial PGN is a hetero-polymer consisting of alternating residues of β-1,4-linked *N*-acetylglucosamine and *N*-acetylmuramic acid to which a peptide chain is attached [24]. Interestingly, *L. monocytogenes* modifies its PGN, with fifty per cent of the muropeptide composition being *N*-deacetylated [25]. We previously reported that a PGN *N*-deacetylase gene, *pgdA*, is responsible for this modification [25]. PGN deacetylation confers resistance to the action of lysozyme, one of the most important and widespread antimicrobial agents of the innate defense system, thus preventing degradation and release of immunostimulants. A strain of *L. monocytogenes* mutated in its ability to alter its PGN, Δ*pgd*A, is sensitive to lysozyme and induces an enhanced IFN-β response in macrophages compared to the isogenic parental strain [25].

The aim of the present study was to decipher the signaling pathways involved in this response to Δ*pgd*A infection. We reveal that IFN-β production in peritoneal macrophages requires TLR2 signaling and the TRIF adapter protein.

## Results

### IFN-β is highly expressed in response to infection with *Listeria ΔpgdA* mutant in a TLR2-dependent manner

**Figure 1:**
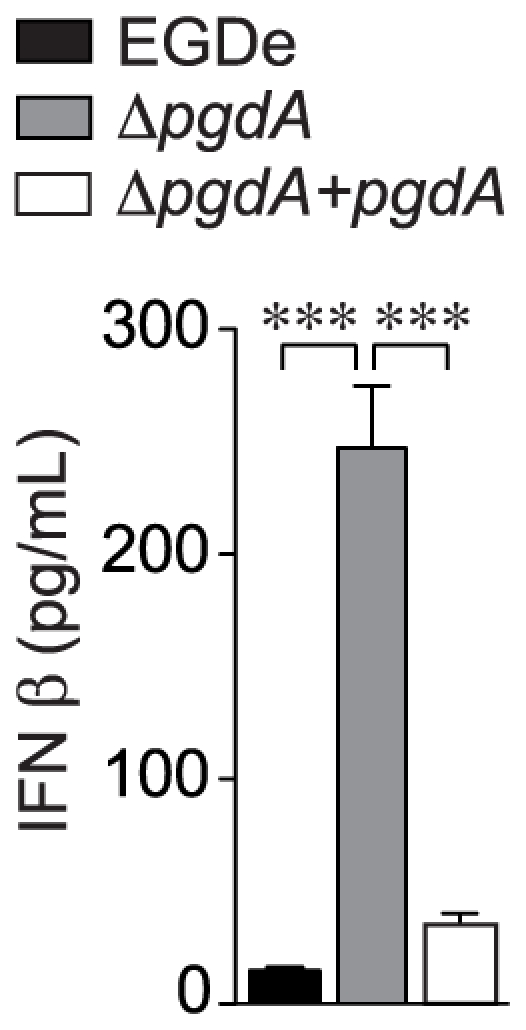
PgdA-dependent IFN-β response to *Listeria* in peritoneal macrophages. PEM from WT C57BL/6J mice were infected with the parental EGDe strain (black bars), the Δ*pgdA* mutant (grey bars) or the complemented Δ*pgdA* strain (white bars). After 7 h of infection, IFN-β levels were measured in supernatants by ELISA. Data are mean ± SD (***, *p*<0.0001, *n* = 5).

A *L. monocytogenes pgdA* mutant induced a much higher IFN-β response than the parental strain [25]. To definitively establish a role for the peptidoglycan deacetylase PgdA in the down-regulation of IFN-β production, we complemented our original *pgdA* mutant with the wild-type gene and we measured IFN-β secretion of peptone elicited peritoneal macrophages (PEM) infected with wild-type EGDe, Δ*pgdA* and a complemented Δ*pgdA* strain (Fig. 1). Inactivation of *pgdA* led to a strong induction of IFN-β secretion in wild-type macrophages. In contrast, the complemented strain did not induce any massive IFN-β secretion, similar to wild-type EGDe. Thus, PgdA directly contributes to down-regulation of IFN-β production.

**Figure 2:**
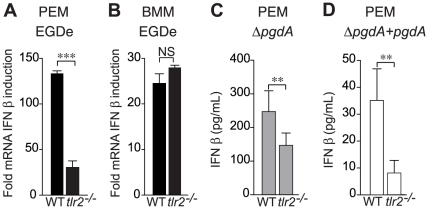
TLR2 is required for PgdA-mediated IFN-β response to *Listeria* in peritoneal but not bone-marrow macrophages. (A) PEM from C57BL/6J or *tlr2^−/−^* mice were infected with the parental EGDe strain. After 4 h of infection, IFN-β induction was measured by qRT-PCR. (B) BMM from C57BL/6J or *tlr2^−/−^* mice were infected with the parental EGDe strain. After 4 h of infection, IFN-β induction was measured by qRT-PCR. Data are mean ± SD (NS, non significant; ***, *p*<0.0001, *n* = 3). PEM from WT C57BL/6J or *tlr2* ^−/−^ mice were infected with the Δ*pgdA* mutant (C) or the complemented Δ*pgdA* strain (D). After 7 h of infection, IFN-β levels were measured in supernatants by ELISA. Data are mean ± SD (**, *p*<0.01, *n* = 5).

Consistent with our previous report measuring secretion of IFN-β protein in PEM, IFN-β mRNA synthesis induced by *L. monocytogenes* infection of PEM required TLR2 (Fig. 2A), while TLR2-deficient BMM showed no impairment in their synthesis of IFN-β mRNA (Fig. 2B). Moreover, IFN-β secretion was strongly reduced in *tlr2* ^−/−^ PEM infected with both the Δ*pgdA* mutant (Fig. 2C) and the complemented Δ*pgdA* strain (Fig. 2D), definitively establishing the TLR2 dependence of IFN-β production.

### IFN-β induction does not require Mal/TIRAP but depends on TRIF

**Figure 3:**
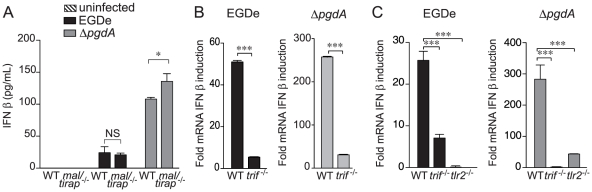
TRIF, but not Mal/TIRAP, is necessary for IFN-β response to *Listeria* in peritoneal macrophages. (A) PEM from WT C57BL/6J or *mal/tirap^−/−^* mice were infected with the parental EGDe strain (black bars), the Δ*pgdA* mutant (grey bars). After 7 h of infection, IFN-β levels were measured in supernatants by ELISA. (B) PEM from C57BL/6J or *trif^−/−^* mice were infected with the parental EGDe strain (black bars) or the Δ*pgdA* mutant (grey bars). After 4 h of infection, IFN-β induction was measured by qRT-PCR. (C) Resident peritoneal macrophages from WT C57BL/6J, *trif* ^−/−^ or *tlr2^−/−^* mice were infected with the parental EGDe strain (black bars), the Δ*pgdA* mutant (grey bars). After 4 h of infection, IFN-β induction was measured by qRT-PCR. Data are mean ± SD (NS, non significant; *, *p*<0.05; ***, *p*<0.0001; *n* = 3–4).

We next analyzed the pathways by which *Listeria* induces IFN-β. Our previous study and the above results strongly suggested the critical involvement of TLR2 [25]. TLR2 signaling depends on Mal/TIRAP and MyD88 adaptor proteins. We had previously shown that MyD88 contributed to full IFN-β induction by *Listeria* [25]. We then compared IFN-β production by wild-type and *mal/tirap* ^−/−^ macrophages infected with EGDe or Δ*pgdA* (Fig. 3A). Surprisingly, production of IFN-β was not decreased in infected macrophages deficient in Mal/TIRAP, indicating that the normal TLR2 adaptor Mal/TIRAP was not required for *Listeria*-mediated induction of IFN-β.

The adapter TRIF is employed by TLRs 3 and 4 to signal through the TBK1-IRF3/7-IFN-β pathway. There is no previous evidence of an association or functional interaction between TRIF and TLR2. In spite of this, the link between TRIF and the IRF pathway on the one hand, and the unusual employment of TLR2 for signaling to the IFN-β gene in PEM on the other suggested the possibility of a role for TRIF. To test this hypothesis we compared induction of IFN-β expression in wild-type and *trif* ^−/−^ PEM or BMM infected with EGDe or Δ*pgdA* strains. IFN-β induction strongly decreased in TRIF-deficient macrophages infected with any of the two *Listeria* strains compared to wild-type PEM, showing the requirement for TRIF (Fig. 3B). In contrast, BMM showed a TRIF-independent IFN-β production (Fig. S1).

The PEM used in our studies are recruited to the peritoneal cavity by injection of the sterile irritant proteose peptone. Hence they differ from BMM not only regarding their anatomical location, but also their partially inflammatory character. To distinguish which of these differences was responsible for the TLR2 and TRIF signaling pathways, we examined IFN-β production by resident PEM. Figure 3C demonstrates a requirement for TLR2 and TRIF by the resident macrophage population. Thus, location to the peritoneal cavity rather than inflammatory character determines the difference in signaling to the IFN-β gene between BMM and PEM.

**Figure 4:**
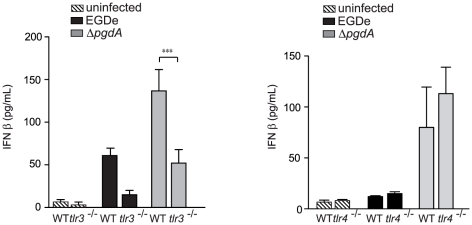
TLR3, but not TLR4, contributes to IFN-β response to *Listeria* in peritoneal macrophages. (A) PEM from WT C57BL/6J or *tlr3* ^−/−^ mice were infected with the parental EGDe strain (black bars), the Δ*pgdA* mutant (grey bars). After 7 h of infection, IFN-β levels were measured in supernatants by ELISA. Data are mean ± SD (n = 3). (B) PEM from WT C57BL/6J or *tlr4* ^−/−^ mice were infected with the parental EGDe strain (black bars), or the Δ*pgdA* mutant (grey bars). After 7 h of infection, IFN-β levels were measured in supernatants by ELISA. Data are mean ± SD (***, *p*<0.0001; *n* = 3).

To examine the role of TLR3, which uses TRIF to trigger IFN-β synthesis, we compared induction of IFN-β in wild-type and *tlr3^−/−^* PEM infected with EGDe or Δ*pgdA* strains. IFN-β production was decreased in TLR3-deficient PEM infected with EGDe or Δ*pgdA* (Fig. 4A). We also compared induction of IFN-β in wild-type and *tlr4^−/−^* PEM infected with EGDe or Δ*pgdA* strains, as TLR4 can mediate TRIF-dependent synthesis of IFN-β. In contrast to TLR3-deficient PEM, TLR4-deficient PEM did not show a decrease in IFN-β response to EGDe or Δ*pgdA* (Fig. 4B). Thus, IFN-β induction in response to *Listeria* infection relies in part on TLR3 and does not require TLR4.

### IFN-β is induced by intracellular bacteria

**Figure 5:**
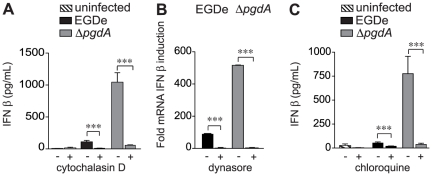
Internalization of bacteria is required for IFN-β response by peritoneal macrophages. (A) PEM from WT C57BL/6J mice were pretreated with 50 µM of cytochalasin D, and left uninfected (hatched bars) or infected with the parental EGDe strain (black bars) or the Δ*pgdA* mutant (grey bars). 7 h post-infection, IFN-β levels were measured in cells supernatants by ELISA. (B) PEM from WT C57BL/6J were treated with 80 µM dynasore. After 4 h of infection with the parental EGDe strain (black bars) or the Δ*pgdA* mutant (grey bars), IFN-β induction was measured by qRT-PCR. (C) PEM from WT C57BL/6J mice were treated with 100 µM chloroquine, and left uninfected (hatched bars) or infected with the parental EGDe strain (black bars) or the Δ*pgdA* mutant (grey bars). 7 h post-infection, IFN-β concentrations were measured in cells supernatants by ELISA. Data are mean ± SD (***, *p*<0.0001, *n* = 3–4).

Induction of IFN-β via TLR2 is no longer an exception. It has recently been shown that vaccinia virus-induced IFN-β production was dependent on TLR2 signaling and it was reported that this was occuring from late endosomes [26]. To investigate if an intracellular localization was also required in the case of *Listeria*, we pretreated cells with cytochalasin D to prevent internalization and measured IFN-β secretion by macrophages infected with EGDe or the Δ*pgdA* mutant (Fig. 5A). In both cases, IFN-β induction was strongly reduced. Thus, internalization is critical for *Listeria*-mediated IFN-β production. We also used dynasore, a dynamin inhibitor and chloroquine, which inhibits endosome acidification, and measured IFN-β induction in macrophages infected with EGDe or the Δ*pgdA* mutant (Fig. 5B–C). IFN-β synthesis was strongly diminished by both dynasore and chloroquine treatments. Together, these results suggest that the TLR2-dependent IFN-β induction is triggered intracellularly.

### IRF3 and IRF7 are essential for IFN-β production in response to *Listeria* infection

**Figure 6:**
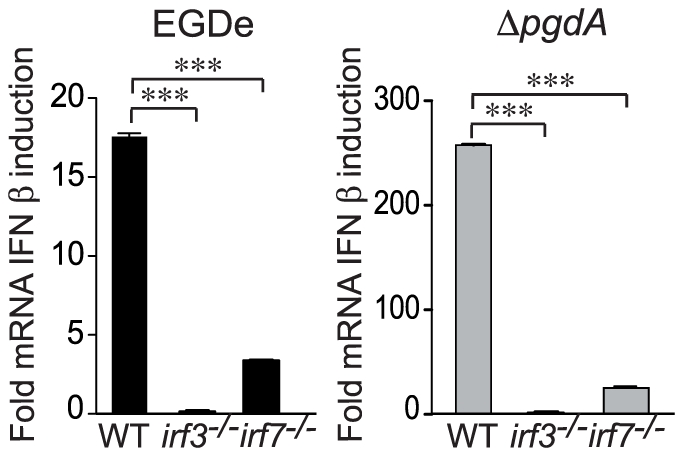
IFN-β response to *Listeria* is mediated by IRF3 and IRF7 in peritoneal macrophages. PEM from WT C57BL/6J, *irf3* ^−/−^ and *irf7* ^−/−^ mice were infected with the parental EGDe strain (black bars) or the Δ*pgdA* mutant (grey bars). 4 h post-infection, mRNA was isolated and the IFN-β induction was measured by qRT-PCR. Data are mean ± SD (***, *p*<0.0001, *n* = 3).

In BMM rapid synthesis of IFN-β is entirely dependent on IRF3, but not on IRF7, whereas in bone marrow-derived myeloid DC IFN-β synthesis requires both IRF3 and IRF7 [27]. We investigated the role of IRF3 and IRF7 in the production of IFN-β by PEM. To this end we infected *irf3* ^−/−^ and *irf7* ^−/−^ macrophages with EGDe or the Δ*pgdA* strains. Inactivation of IRF3 totally abrogated IFN-β mRNA induction in response to both strains (Fig. 6). IFN-β induction in IRF7-deficient macrophages was also strongly affected highlighting the important role of both transcription factors in response to *Listeria* infection (Fig. 6). PEM thus resemble bone marrow-derived myeloid DC, not BMM, in relation to their IRF requirement for *Listeria*-mediated IFN-β synthesis.

**Figure 7:**
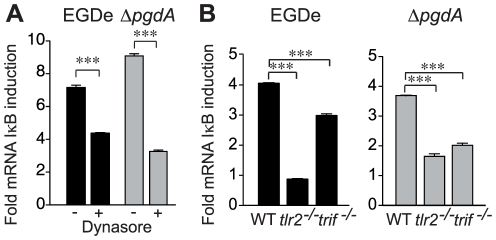
Bacterial internalization and NF-κB are required for TLR2 and TRIF-dependent IFN-β response in peritoneal macrophages. (A) PEM from WT C57BL/6J mice and pretreated with dynasore were infected with the parental EGDe strain (black bars) or the Δ*pgdA* mutant (grey bars). 4 h post-infection, IκB induction was measured by qRT-PCR. (B) PEM from WT C57BL/6J, *tlr2* ^−/−^ and *trif* ^−/−^ mice were infected with the parental EGDe strain (black bars) or the Δ*pgdA* mutant (grey bars). 4 h post-infection, mRNA was isolated and IκB induction was measured by qRT-PCR. Data are mean ± SD (***, *p*<0.0001, *n* = 3).

In addition to IRF3/7, NFκB contributes to the formation of the IFN-β enhanceosome [20], [28]. We therefore examined the involvement of the NFκB pathway by measuring induced synthesis of an NFκB-dependent mRNA. IκB is an NFκB-dependent gene and thus a read-out for NFκB activation in response to *Listeria* infection. We measured the induction of IκB expression in PEM infected with EGDe or the Δ*pgdA* mutant. Both strains induced IκB expression and this required internalization as treatment with dynasore reduced the level of IκB induction (Fig. 7A). Degradation of the IκB protein was examined in PEM infected with EGDe by immunoblot using anti-IκB antibodies. IκB level was reduced rapidly after infection of wild-type PEM (Fig. S2A). In contrast, IκB degradation was not observed in *tlr2* ^−/−^ PEM infected with *Listeria* (Fig. S2B). Infection of wild-type, *tlr2* ^−/−^and *trif* ^−/−^ macrophages with EGDe or Δ*pgdA* showed that both TLR2 and the adaptor were required for full induction of IκB mRNA in response to EGDe and Δ*pgdA* strains (Fig. 7B). These results suggest that TLR2 and TRIF contribute to NFκB activation. The comparison between EGDe and Δ*pgdA* strains showed that both caused similar magnitudes of IκB mRNA synthesis. Thus, the activation of NFκB by *Listeria* is independent of PgdA, suggesting that the increased IFN-β production after infection with Δ*pgdA* relies on activation of other transcription factors such as IRFs.

### Nucleic acids released intracellularly are critical for IFN-β induction

TLR2 or TRIF deficiency strongly reduced, but did not completely shut off IFN-β synthesis. This suggested a potential contribution of intracellular, nucleic acid-dependent pathways to IFN-β synthesis, particularly after infection with Δ*pgdA*. We therefore examined whether these pathways are able to signal in PEM.

**Figure 8:**
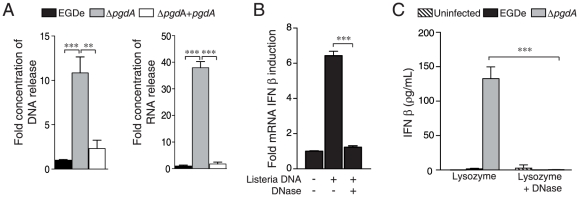
*Listeria* nucleic acids trigger IFN-β production. (A) The parental EGDe (black bars) Δ*pgdA* (grey bars) and complemented Δ*pgdA* strain (hatched bars) were incubated with lysozyme. The amount of DNA and RNA released after treatment was quantified by spectrophotometry. (B) THP-1 macrophages were transfected with DNA from the Δ*pgdA* mutant, pretreated or not with DNase, and IFN-β induction was determined using the HEK-blue assay. (C) The parental EGDe strain (black bars) or the Δ*pgdA* mutant (grey bars) were incubated with lysozyme. PEM were transfected with bacterial lysates, pretreated with DNase or not treated, and IFN-β production was quantified in cells supernatants 24 h after transfection by ELISA. Data are mean ± SD (**, *p*<0.01, n = 3; ***, *p*<0.0001, *n* = 3).

Since inactivation of PgdA increases *Listeria* sensitivity to peptidoglycan-targeting antimicrobials such as lysozyme, and thus induces bacterial degradation, we measured the DNA and RNA released by EGDe and Δ*pgdA* strains following lysozyme exposure. As expected, Δ*pgdA* released significantly higher amounts of DNA and RNA than wild-type and complemented Δ*pgdA* strains, raising the possibility that both DNA and RNA could be involved in IFN-β production (Fig. 8A). We thus measured IFN-β induction in THP1 macrophages transfected with *Listeria* DNA, either undigested or treated with DNase. Intact but not DNase-treated DNA significantly induced IFN-β (Fig. 8B). Macrophages were then transfected with lysozyme-digested EGDe or Δ*pgdA*, either untreated or digested with DNase. Treatment with DNase significantly reduced IFN-β production (Fig. 8C). Taken together, these results show that *Listeria* DNA can induce IFN-β, strongly indicating that destruction of Δ*pgdA* bacteria intracellularly activates DNA sensors.

## Discussion

We had recently reported that a PGN modification involving a *N*-deacetylase gene, *pgdA*, was playing a key role in *L. monocytogenes* virulence [25]. A Δ*pgdA* strain of *L. monocytogenes* which is unable to modify its PGN, was shown to be extremely sensitive to the bacteriolytic activity of lysozyme, normally found within macrophage vacuoles and its virulence was strongly attenuated [25]. Furthermore, this mutant induced a much higher TLR2-dependent IFN-β response than the parental strain [25]. We hypothesised that this unconventional IFN-β response induced by the *pgdA* mutant was due to an enhanced accessibility of bacterial cell wall components to TLR2. Here we have shown that IFN-β production requires bacterial internalization and is triggered by Mal/TIRAP-independent pathways which involve TLR2, TRIF, IRF3 and IRF7.

It was surprising to see a role for TLR2, as, based on results in BMM and epithelial cells, type I IFNs production is usually not known to result from TLR2 signaling [5]–[8]. Classical TLR2 signaling leads to NF-κB-dependent production of inflammatory cytokines [21]. However, in support of an unconventional role for TLR2, recent studies reported roles for TLR2-dependent induction of IFN-β in response to vaccinia virus or synthetic ligands [26], [29]. In the vaccinia virus study, a specific inflammatory monocyte population -Ly6C^hi^- was shown to be the source of IFN-β [26]. In the present study we show that TLR2-dependent IFN-β synthesis is a property of both resident and recruited inflammatory PEM. Furthermore, the two previous studies documented that TLR2 activation of type I IFN responses to TLR ligands occurs within intracellular compartments, and that TLR2 signals from the phagosome in response to viral infection or synthetic TLR2 ligands [26], [29]. These results challenged the view that TLR2 signals solely from the plasma membrane. In our experiments, pre-treatment of PEM with either cytochalasin D, an inhibitor of actin polymerization and thus internalization, dynasore, an inhibitor of the endocytic effector dynamin, or chloroquine, which inhibits endosome acidification [30], [31], significantly impaired the induction of IFN-β following *Listeria* infection, strongly suggesting that phagocytosis of *L. monocytogenes* and intracellular location of TLR2 trigger this response. These observations also correlate with our early hypothesis that the inflammatory response induced by Δ*pgdA* is due to an enhanced release or accessibility of bacterial cell wall components to TLR2.

Induction of the IFN-β gene was independent of the TLR adapter Mal/TIRAP, but, unexpectedly required the TLR3/4 adapter TRIF. *Francisella tularensis* has recently been shown to signal through TLR2 from the phagosome in a Mal/TIRAP independent manner [32], and it was shown that Mal/TIRAP is dispensable in TLR2 signaling at high concentrations of ligands [33]. Thus our study reinforces the view that TLR2 can act independently from Mal/TIRAP. In addition our report suggests a synergy between a TLR2 pathway and TRIF, an adapter previously known to trigger the synthesis of pro-inflammatory cytokines and type I IFNs upon engagement of TLR3 and TLR4. TLR3 is known to bind viral dsRNA to induce secretion of type I IFN and lead to control of viral infections [3], [34], [35]. To our knowledge *Chlamydia muridarum* is the only bacterium reported to induce a TLR3-dependent IFN-β response specifically in murine oviduct epithelial cells [36]. We tested whether the dual requirement for TLR2 and TRIF resulted from a functional or physical interaction between TLR2 and TLR3. In fact, IFN-β production was reduced in TLR3-deficient macrophages, but significantly less so than in *trif−/−* PEM. Therefore, there is no evidence for a putative TLR2/TLR3 interaction. Another possibility to incorporate TRIF into the pathway stimulated in PEM by *Listeria* would be a cooperation of TLR2 and TLR4. This was ruled out by showing that *Listeria*-infected *tlr4^−/−^* PEM produced a similar amount of IFN-β as their wild-type counterparts. TRIF could possibly orchestrate an additional pathway. Along these lines, TRIF has recently been shown to be required for IFN-β synthesis by dendritic cells upon activation of the cytosolic receptor complex DDX1/DDX21/DDX36 by viral RNA [19].

Engagement of TLRs by various microbe-associated molecular patterns induces activation and translocation to the nucleus of NF-κB, IRF3, IRF7 and/or activator protein-1 (AP-1), which collaborate to induce transcription of type I IFNs [37]. We addressed the role of these transcriptional activators in the IFN-β response to wild-type *Listeria* and Δ*pgdA*, and revealed that inactivation of IRF3 totally abrogated this response to both strains while IFN-β induction was significantly but not totally impaired in IRF7-deficient macrophages, indicating that both of these transcription factors are required for induction of IFN-β following infection with *L. monocytogenes*. We also assessed the involvement of NF-κB in this response using induction of the IκB gene as a readout. We observed an induction of IκB expression in macrophages which was similar after infection with EGDe or Δ*pgdA*. Thus, activation of NF-κB by *Listeria* is independent of PgdA, strongly suggesting that the elevated IFN-β production by the Δ*pgdA* mutant mostly relies on IRF3.

The increased IFN-β response to the Δ*pgdA* strain probably results from the fact that within the phagosome, its lysozyme-sensitive cell wall is degraded, releasing PAMPs able to interact with TLR2 and other PRRs, including cytoplasmic ones. As recent studies have highlighted novel DNA-sensing pathways in the induction of type I IFNs [9], [14]–[17], [38], we thus also investigated the involvement of bacterial nucleic acids in the IFN-β induction, Firstly, we showed that inactivation of PgdA, which confers a higher susceptibility to lysozyme, leads to increased release of DNA. We then showed that DNA from *L. monocytogenes* can induce IFN-β expression in PEM, suggesting that this macrophage population employs cytoplasmic nucleic acid sensing similar to macrophages or macrophage lines derived from different anatomical locations [9], [38]. Which -if any- of the recently described nucleic acid sensors are used by PEM for the recognition of *Listeria* DNA remains subject to future investigation. Nevertheless, other bacterial components could participate in IFN-β production upon infection with the Δ*pgdA* mutant. For example, the second messenger molecule cyclic diadenosine monophosphate (c-di-AMP), was shown to be secreted by *Listeria* multidrug efflux pumps triggering type I IFN response [10] and could be involved in the process.

In conclusion, this study describes a novel mechanism leading to induction of type I IFNs in which intracellular sensing plays an important role, ultimately showing how these different recognition pathways can synergise to induce innate immune responses which are required to control infection. In this regard cooperation between TLR2 and TRIF may reflect the need for convergence of the NF-κB and IRF pathways at the IFN-β promoter, with TLR2 being responsible mainly for NF-κB activation and TRIF being instrumental for activation of IRF3 and IRF7. By employing the strategy of PGN modification, *L. monocytogenes* can avoid immune detection by TLR and evade the innate immune response, thus enabling the infectious process to occur. It is important to recall that *pgdA* orthologs are found in other pathogenic bacteria, such as *Streptococcus pneumoniae*, *Bacillus cereus*, *Bacillus anthracis* and *Helicobacter pylori*, strongly suggesting that PGN N-deacetylation is a general mechanism evolved by microbes to escape from pattern recognition receptor-mediated immune recognition [39]–[42].

## Materials and Methods

### Bacterial strains and growth conditions

*L. monocytogenes* EGDe (BUG1600, ATCC BAA-679), *L. monocytogenes* isogenic mutant Δ*pgdA* (BUG2288, [25]) and *L. monocytogenes* Δ*pgdA* complemented strain (BUG2382) were grown in brain heart infusion (BHI, Oxoid), aerobically at 37°C and 200 rpm.

### Construction of *L. monocytogenes ΔpgdA* complemented strain

A DNA fragment containing the *pgdA* gene (*lmo0415*) and its promoter was generated by PCR using oligonucleotides lmo0415-1 (5′-AAGGATCCCACAATATGTTAGTTTTCAGGGG-3′) and lmo0415-2 (5′-AAGGATCCTTATTTCACCATTCTTGAATCTG-3′). The fragment was integrated into pCR-Blunt-II-TOPO (Invitrogen) and the construct was verified by sequencing. After digestion of the construct by *Bam*HI, the fragment was purified on agarose gel and cloned into the integrative vector pPL2 [43], previously digested by *Bam*HI, constructing pOD98. The pOD98 was electroporated into Δ*pgdA* at 2,500 V, 200 Ω and 25 µF. Transformants were selected at 37°C on BHI agar containing chloramphenicol (7 µg/mL). The presence of the *pgdA* gene in the complemented strain was confirmed by PCR using oligonucleotides lmo0415-1 and lmo0415-2.

### Ethics statement

Mice were used for obtaining peptone-elicited peritoneal macrophages, resident peritoneal macrophages and bone marrow-derived macrophages. Animal experiments were performed in accordance with protocols approved by the Animal Experimentation Ethics Committee of the Institut Pasteur (permit #03-49) and following Austrian law in accordance with protocols approved by the Ethics Committee of the University of Veterinary Medicine, Vienna (#GZ680 205/67-BrGt/2003).

### Isolation and culture of murine peptone-elicited peritoneal macrophages (PEM)

PEM were isolated from 7 to 10 week-old C57BL/6J and genetically-matched *tlr2* ^−/−^, *tlr3* ^−/−^, *tlr4* ^−/−^, *mal/tirap* ^−/−^, *trif* ^−/−^, *irf3* ^−/−^ and *irf7* ^−/−^ mice as previously described [44]. The percentage of macrophages was determined by flow cytometry using CD11b (1∶100, eBiosciences) and F4/80 (1∶100, eBiosciences) antibodies. More than 90% of the cells were macrophages. PEM were seeded onto 6-well plates at a concentration of 2×10^6^ cells per well in DMEM (PAA) supplemented with 10% FCS, 10% L929 conditioned medium (LCM) and 1% penicillin-streptomycin or RPMI-1640 (Gibco) supplemented with 10% FBS and 1% penicillin-streptomycin.

### Isolation of resident peritoneal macrophages

Resident macrophages were isolated from 6 to 8 week-old C57BL/6J and genetically-matched *tlr2* ^−/−^, *trif* ^−/−^ mice by washing the peritoneum twice with 10 mL DMEM (PAA) supplemented with 10% FBS, 10% LCM and 1% penicillin-streptomycin. Harvested cells were centrifuged at 300 *g* for 5 minutes and resuspended in complete medium. The percentage of macrophages was determined by flow cytometry analysis as above. Cells were seeded onto 6-well plates (Nunc) at a concentration of 2×10^6^ cells per well.

### Isolation of bone marrow-derived macrophages

Tibia and femur from 6 to 8 week-old C57BL/6J and genetically-matched *tlr2* ^−/−^, *trif* ^−/−^ mice were collected in ice cold PBS. Bones were sterilized with 70% ethanol and flushed with a 25-G needle using cold DMEM supplemented with 10% FCS, 10% LCM and 1% penicillin-streptomycin. Cells were seeded onto 6-well plates (Nunc) at a concentration of 10^6^ cells per well and incubated at 37°C with 5% CO_2_. After 4 days, complete medium was added and cells were split at a ratio of 1∶2. After 8 days, macrophages were fully differentiated.

### Culture of human THP-1-derived macrophages and HEK-blue type I IFN cells

Human acute monocytic leukemia THP-1 cells (ATCC TIB202) were maintained in RPMI-1640 supplemented with 10% FBS and 1% penicillin-streptomycin. Cells were seeded onto a 24-well plate at a concentration of 4×10^5^ cells per well in antibiotic-free media supplemented with 12.5 ng/mL phorbol myristate acetate and incubated for 24 h at 37°C with 5% CO_2_. Differentiation was determined to be successful upon formation of a confluent adherent monolayer. HEK-blue type I IFN cells (Invivogen) were grown in DMEM supplemented with 10% FBS and 1% penicillin-streptomycin. Cells were seeded at a concentration of 5.6×10^4^ cells per well onto a 96-well plate.

### Macrophage infection assays

For cytokine analysis, macrophages were infected with *Listeria* strains at MOI 10∶1, centrifuged at 300 *g* for 2 min and incubated at 37°C for 15 min. Following phagocytosis, monolayers were washed twice followed by incubation in RPMI-1640 supplemented with 10% fetal bovine serum (FBS) and gentamicin (20 µg/mL). Supernatants were collected at various time points, for detection of IFN-β by ELISA. For transcript analysis, macrophages were infected with *Listeria* strains at MOI 20∶1 and incubated at 37°C for 1 h to allow phagocytosis. Monolayers were washed and incubated in DMEM supplemented with 10% FCS and gentamicin (5 µg/mL). After 2 h, medium was changed to DMEM supplemented with 10% FCS and gentamicin (1 µg/mL). Cells were lysed at various time points and RNA collected for qPCR analysis.

### Inhibition assays

For inhibition of bacterial internalization, cell monolayers were pretreated either for 2 h with 100 µM cytochalasin-D (Sigma-Aldrich), or 30 min with 80 µM dynasore (Sigma-Aldrich) or 30 min with 100 µM chloroquine (Sigma-Aldrich) prior to infection assays.

### DNA isolation and transfection assays

*Listeria* were grown overnight in BHI at 37°C and cultures were centrifuged at 8000 *g* for 5 min. Bacterial pellets were resuspended in 75 µg/mL lysozyme and incubated at 37°C for 1 h. DNA was then extracted using the DNeasy blood and tissue kit (Qiagen) and quantified by spectrophotometry (Nanodrop). For transfection assays, THP-1 macrophages were transfected with 200 ng/mL DNA with 2% lipofectamine 2000 (Invitrogen) and incubated for 24 h. Following incubation, supernatants were collected for IFN-β analysis. For pretreatment of DNA with DNase, DNase was added at final concentration of 100 µg/mL for 45 min at 37°C.

### Lysozyme digestion, quantification of nucleic acids release and identification of *Listeria* PAMPs

Bacterial cultures were treated with 10 µg/mL lysozyme, a concentration leading to lysis of Δ*pgdA* but not EGDe, and incubated at 37°C and 200 rpm for 1 h. Following lysozyme treatment, lysed bacterial cultures were centrifuged at 5000 rpm during 10 min. Two types of experiments were performed on supernatants. First, nucleic acid release was quantified. DNA was purified using the Qiagen DNeasy blood and tissue kit omitting lysis steps and quantified by spectrophotometry (Nanodrop). RNA was purified using Qiagen RNeasy kit and quantified by spectrophotometry (Nanodrop). Data shown are representatives of at least three independent experiments. Second, 100 µL of each supernatants were treated by DNase during 30 min at 37°C. Enzymes were inactivated and treated- or untreated-supernatants were transfected in PEM. 8 h after transfection, supernatants of cells were recovered and the IFNβ was quantified.

### Detection of type I IFN by ELISA and HEK-blue type I IFN cell assay

Murine IFN-β production was detected in macrophage supernatants by ELISA according to the manufacturer's procedure (PBL Biomedical Laboratories). For the HEK-blue type I IFN assay, supernatant from THP-1 macrophage assays was collected and 20 µL added onto HEK-blue type I IFN cells plated in 96-well plates, which were incubated at 37°C overnight. Supernatant from HEK-blue cells was collected and 40 µL added to 160 µL of Quanti-blue reagent (Invivogen) for 20 min at 37°C. The colorimetric reaction was measured at 625 nm on a plate reader. Data was normalised against absorbance for the untreated cells and plotted as relative fold increases. Data shown are representatives of at least three independent experiments.

### Detection of IkB by immunoblot

PEM from WT or *tlr2* ^−/−^ C57BL/6J mice were infected with EGDe. Cells were lysed 0, 0.5, 1, 1.5, 2, 2.5, or 3 h post-infection. IκB and tubulin were detected in lysates by immunoblotting using anti-IκB (Santa Cruz, 1∶100) and anti-α-tubulin (Sigma, 1∶5000) antibodies.

### RNA isolation for quantitative real-time PCR

RNA preparation was performed using NucleoSpin RNA II kit (Macherey-Nagel) according to the manufacturer's instructions. Quantitative real-time PCR was performed on a Mastercycler EP realplex S (Eppendorf). Primers for HPRT (housekeeping gene control), IFNβ and IκBα mRNA expression were as follows: HPRT forward GTTGGATACAGGCCAGACTTTGTTG, HPRT reverse GAGGGTAGGCTGGCCTATTGGCT, IFNβ forward 5′-TCAGAATGAGTGGTGGTTGC-3′, IFNβ reverse 5′-GACCTTTCAAATGCAGTAGATTCA-3′; IκBα forward 5′-GCAATTTCTGGCTGGTGGG-3′, IκBα reverse 5′GATCCGCCAGGTGAAGGG-3′. Data shown are representatives of at least three independent experiments.

### Statistical analysis

Results are expressed as means of at least three values, with error bars representing standard deviations. Student's *t* tests were performed to determine statistical significance where ^*^ indicates *P*<0.05, ^**^ indicates *P*<0.01 and ^***^ indicates *P*<0.0001.

## Supporting Information

Figure S1 **TRIF is not required for IFN-β response to** ***Listeria*** **in bone marrow macrophages.** BMM from C57BL/6J or *trif^−/−^* mice were infected with the parental EGDe strain (black bars) or the Δ*pgdA* mutant (grey bars). After 4 h of infection, IFN-β induction was measured by qRT-PCR. Data are mean ± SD (NS, non significant, *n* = 3).
(EPS)

Figure S2 **TLR2 is required for optimal activation of NF-κB.** (A) PEM from WT C57BL/6J mice were infected with EGDe. Cells were lysed 0, 0.5, 1, 1.5, 2, 2.5, or 3 h post-infection. Activation of NF-κB was measured by determination of IκB degradation relative to tubulin following immunodetection. (B) PEM from *tlr2* ^−/−^ mice were infected with EGDe. Cells were lysed 0, 0.5, 1, 1.5, 2, 2.5, or 3 h post-infection. Activation of NF-κB was measured by determination of IκB degradation relative to tubulin following immunodetection.
(EPS)
